# Impact of opioid analgesics on survival in cancer patients receiving immune checkpoint inhibitors

**DOI:** 10.1007/s00520-024-08681-2

**Published:** 2024-06-27

**Authors:** Gozde Kavgaci, Deniz Can Guven, Yunus Kaygusuz, Ece Karaca, Omer Dizdar, Saadettin Kilickap, Sercan Aksoy, Mustafa Erman, Suayib Yalcin

**Affiliations:** 1 Department of Medical Oncology, Hacettepe University Cancer Institute, Ankara, Turkey; 2 Department of Internal Medicine, Hacettepe University Faculty of Medicine, Ankara, Turkey; 3 Department of Medical Oncology, Liv Hospital, Ankara, Turkey; 4 Department of Medical Oncology, Istinye University Faculty of Medicine, Istanbul, Turkey

**Keywords:** Immune checkpoint inhibitors, Opioid analgesics, Cancer pain management, Survival

## Abstract

**Purpose:**

This study aimed to assess the effects of concurrent opioid analgesic (OA) use with immune checkpoint inhibitors (ICIs) on progression-free survival (PFS) and overall survival (OS).

**Methods:**

In this observational retrospective study, we included advanced cancer patients who received ICIs at Hacettepe University Hospital’s Department of Medical Oncology between June 2018 and January 2023.

**Results:**

Our study included 375 recurrent or metastatic cancer patients treated with ICIs in the first, second line, or beyond. There were no significant differences between the OA-treated and OA-untreated groups regarding median age, age group, gender, primary tumor location, ICI type, or the presence of baseline liver and lung metastases. However, the OA-treated group exhibited a significantly higher proportion of patients who had received three or more prior treatments before initiating ICIs (*p* = 0.015). OA-Untreatment was significantly correlated with prolonged mPFS (6.83 vs. 4.30 months, HR 0.59, 95% CI 0.44–0.79, *p* < 0.001) and mOS (17.05 vs. 7.68 months, HR 0.60, 95% CI 0.45–0.80, *p* < 0.001).

**Conclusions:**

Our study demonstrates an association between the concurrent use of OAs and reduced OS and PFS in patients treated with ICIs. While OA treatment serves as a surrogate marker for higher disease burden, it may also suggest a potential biological relationship between opioids and immunotherapy efficacy.

## Introduction

The field of oncology has experienced an evolutionary change with the introduction of immune checkpoint inhibitors (ICIs), which have shown significant improvements in overall survival (OS) for various types of cancer [1]. These developments have introduced a new era in cancer treatment with its own challenges. The emergence of drug-drug interactions (DDIs) poses novel challenges in optimizing the concurrent use of medications alongside ICIs. Antibiotics, proton pump inhibitors, and steroids have all been identified as potential influences on the prognosis of patients receiving ICIs [2, 3].

According to the World Health Organization’s (WHO) recommendations, non-steroidal anti-inflammatory drugs (NSAIDs) for mild pain are followed by potent opioid analgesics (OAs) for severe chronic pain [4]. This analgesic ladder is tailored to the intensity of the pain. Within the realm of cancer care, OA treatment is inevitable for patients dealing with moderate to severe pain. However, the influence of OAs on the efficacy of ICIs remains a subject characterized by uncertainty, necessitating the presence of strong clinical data. Preclinical investigations have suggested that OAs might facilitate tumor progression and metastasis due to the increased expression of opioid receptors within various tumor types [5]. Furthermore, OAs can disturb the composition of the intestinal microbiota and increase the presence of regulatory T cells (Tregs), resulting in impairment of the immune system. Retrospective cohort studies have indicated that the use of OAs may correlate with less favorable prognoses among patients treated with ICIs [6–9].

Our study aims to reveal the relationship between concurrent ICI and OA treatment with a focus on their collective impact on oncological outcomes.

## Methods

### Study population

This observational, retrospective study recruited adult patients with recurrent or metastatic cancer treated with anti-PD-1 or anti-PD-L1 monoclonal antibodies for any cancer type between June 2018 and January 2023 in Hacettepe University Hospital’s Department of Medical Oncology. Except for patients treated within clinical trials or expanded access programs, all individuals treated during the specified time periods were included in the analysis. We excluded patients receiving ICI as neoadjuvant or adjuvant treatment. For OA treatment, we included patients using morphine, oxycodone, oxymorphone, hydromorphone, fentanyl, codeine, or tramadol for cancer pain management, in accordance with the cancer pain management guidelines of our institution. Detailed patient information was collected using electronic medical records, including demographic features, Eastern Cooperative Oncology Group (ECOG) performance status (PS), OA treatment and type, primary tumor site, the sites of metastases, treatment regimen, previous lines of chemotherapy or targeted therapy, death date, and last follow-up visit. The study was conducted in accordance with the Declaration of Helsinki, approved by the Ethics Committee of Hacettepe University, and exempted from informed consent due to its retrospective nature.

### Statistical analysis

Descriptive statistics were presented as the median, minimum, and maximum values for continuous variables, and frequency and percentages for categorical variables. Independent group comparisons were made using independent samples *t*-tests and chi-square tests for continuous and categorical variables, respectively. The OS time was defined as the period from treatment initiation to the last follow-up and/or death, and progression-free survival (PFS) time was defined as the period between treatment initiation to disease progression and/or death. Survival analyses were conducted using Kaplan–Meier analyses, and comparisons of survival times between prognostic subgroups were done using the log-rank test. Multivariate analyses were conducted by the Cox-regression analyses and hazard ratios were calculated together with 95% confidence intervals (CI). All statistical analyses were performed in SPSS, version 25.0 (IBM Inc., Armonk, NY, USA), and a type-I error level of 5% (*p* < 0.05) was considered as the threshold limit for statistical significance.

## Results

### Patient characteristics

Our study included a total of 375 patients with recurrent or metastatic cancer who received treatment with anti-PD-1 or anti-PD-L1 monoclonal antibodies, whether in the first, second line or beyond. The median age of the patients was 62 years (min–max 18–92), with 219 (58.4%) patients being younger than 65 years old. Approximately half of the patient population had an ECOG PS of 0 (190 patients, 50.7%). The most frequently used ICI was nivolumab (302 patients, 80.5%), followed by atezolizumab (45 patients, 12%), pembrolizumab (27 patients, 7.2%), and avelumab (1 patient, 0.3%).

There were no significant differences between the OA-treated and OA-untreated groups in terms of median age, age group, gender, primary tumor location, ICI type, or the presence of baseline liver and lung metastases. However, the OA-treated group had a significantly higher number of patients who had received three or more prior treatments before ICI initiation compared to the OA-untreated group (*p* = 0.015). A summary of the comparison of baseline characteristics between the OA-treated and OA-untreated groups is presented in Table 1.

**Table 1:** Comparison of baseline characteristics in the OA-treated and OA-untreated groups

		OA-treated group (*n* =)	OA-untreated group (*n* =)	*p*-value
Median age (min–max)		62 (20–88)	62 (18–92)	0.476
Age group	< 65 years of age	96 (59.6%)	123 (57.5%)	0.751
	≥ 65 years of age	65 (40.4%)	91 (42.5%)	
Sex	Female	54 (33.5%)	70 (32.7%)	0.912
	Male	107 (66.5%)	144 (67.3%)	
Primary tumor	NSCLC	39 (24.2%)	42 (19.6%)	0.639
	RCC	32 (19.9%)	47 (22%)	
	Melanoma	15 (9.3%)	48 (22.4%)	
	HNC	18 (11.2%)	20 (9.3%)	
	TCC	10 (6.2%)	9 (4.2%)	
	SCLC	11 (3.1%)	11 (4.2%)	
	Sarcoma	4 (2.5%)	6 (2.8%)	
	HCC	5 (3.1%)	5 (2.3%)	
	Breast	5 (3.1%)	5 (2.3%)	
	MPM	7 (4.3%)	2 (0.9%)	
	HL	1 (0.9%)	1 (1.1%)	
	Other	20 (12.4%)	20 (9.3%)	
Line of treatment before ICI	0–2	115 (74.2%)	167 (85.2%)	**0.015**
	3 or more	40 (25.8%)	29 (14.8%)	
Immunotherapy agent	Nivolumab	126 (78.3%)	176 (82.2%)	0.488
	Atezolizumab	20 (12.4%)	25 (11.7%)	
	Pembrolizumab	14 (11.6%)	13 (6.1%)	
	Avelumab	1 (0.4%)	0 (0%)	
Liver metastasis	Absent	118 (73.3%)	162 (75.7%)	0.522
	Present	43 (26.7%)	52 (24.3%)	
Lung metastasis	Absent	82 (50.9%)	108 (50.5%)	0.929
	Present	79 (49.1%)	106 (49.5%)	

*RCC* renal cell carcinoma, *NSCLC* non-small cell lung cancer, *HNC* head and neck cancer, *MPM* malignant pleural mesothelioma, *TCC* transitional cell carcinoma, *SCLC* small cell lung cancer, *HL* Hodgkin lymphoma, *HCC* hepatocellular carcinoma, *OA* opioid analgesic

### Outcomes

**Fig. 1:**
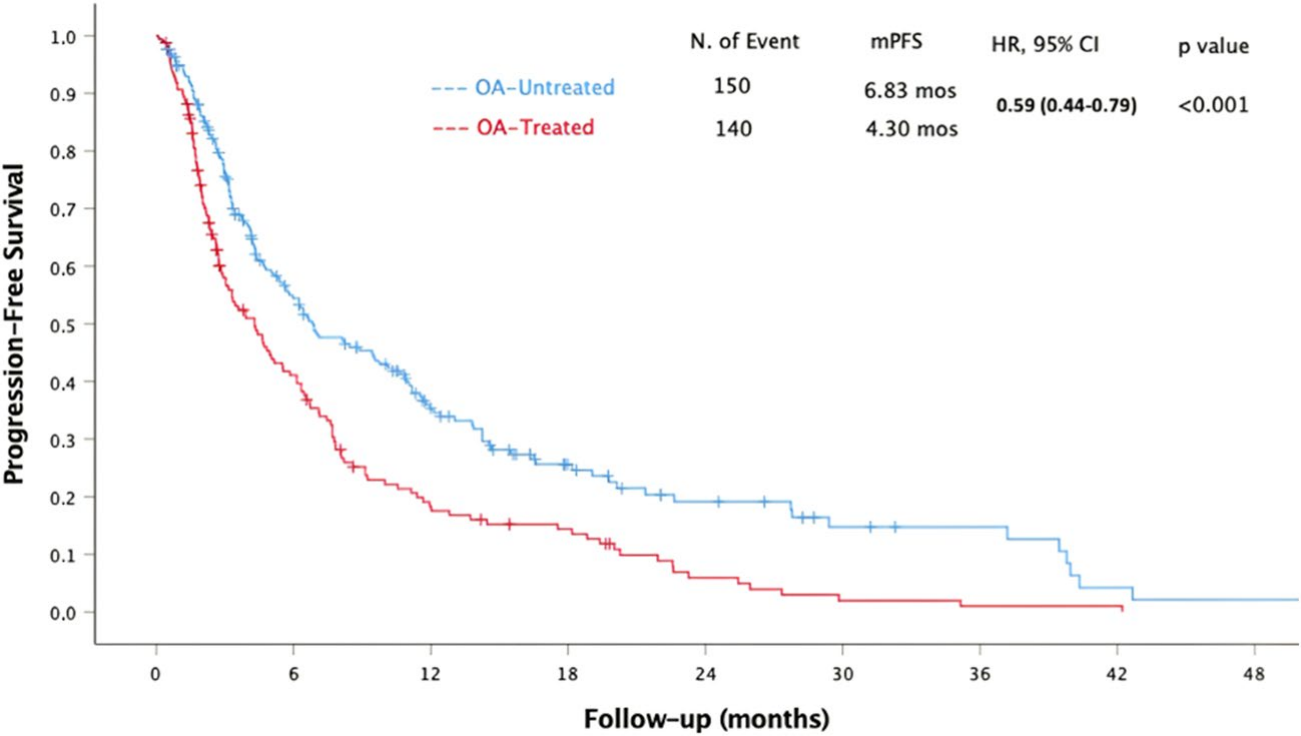
The association between OA treatment and progression-free survival

In our study, the median PFS (mPFS) was 5.71 months (95% CI 4.69 to 6.73) with 290 progression events. Univariate analyses revealed that OA-untreated patients had significantly longer mPFS (*p* < 0.001), as did patients with an ECOG PS equal to 0 (*p* = 0.011) and patients with lactate dehydrogenase (LDH) levels within or below the upper limit of the normal range (ULN) (*p* < 0.001). Univariate analysis demonstrated that mPFS was significantly longer in patients with renal cell carcinoma (RCC) (HR 0.42, 95% CI 0.27–0.65, *p* < 0.001), non-small cell lung cancer (NSCLC) (HR 0.57, 95% CI 0.38–0.84, *p* = 0.05), and small cell lung cancer (SCLC) (HR 0.57, 95% CI 0.32–0.87, *p* = 0.013) compared to melanoma. Multivariate analyses also showed significantly longer mPFS in OA-untreated patients (*p* < 0.001) and in patients with LDH levels within or below the ULN (*p* = 0.016). Detailed results of the univariate and multivariate analyses for PFS are presented in Table 2. OA-Untreatment was significantly associated with longer mPFS (6.83 vs. 4.30 months, HR 0.59, 95% CI 0.44–0.79, *p* < 0.001), as illustrated in Fig. 1.

**Table 2:** Univariate and multivariate analyses for progression-free survival

	Univariate analysis			Multivariate analysis		
	Hazard ratio	95% CI	*p* value	Hazard ratio	95% CI	*p* value
ECOG PS 0 vs. ≥ 1	0.70	0.53–0.92	**0.011**	0.85	0.65–1.10	0.224
< vs. ≥ 65 years of age	0.87	0.67–1.14	0.345			
LDH N vs. > ULN	0.54	0.41–0.72	** < 0.001**	0.73	0.56–0.94	**0.016**
Primary tumor	0.42	0.27–0.65	**0.042**	0.57	0.21–1.48	0.216
Line of treatment before ICI (3 or more)	0.96	0.70–1.23	0.643			
OA-untreated vs. OA-treated	0.59	0.44–0.79	** < 0.001**	0.62	0.49–0.80	** < 0.001**

*ECOG PS* Eastern Cooperative Oncology Group performance score, *LDH* lactate dehydrogenase, *ULN* upper limit of the normal range

**Fig. 2:**
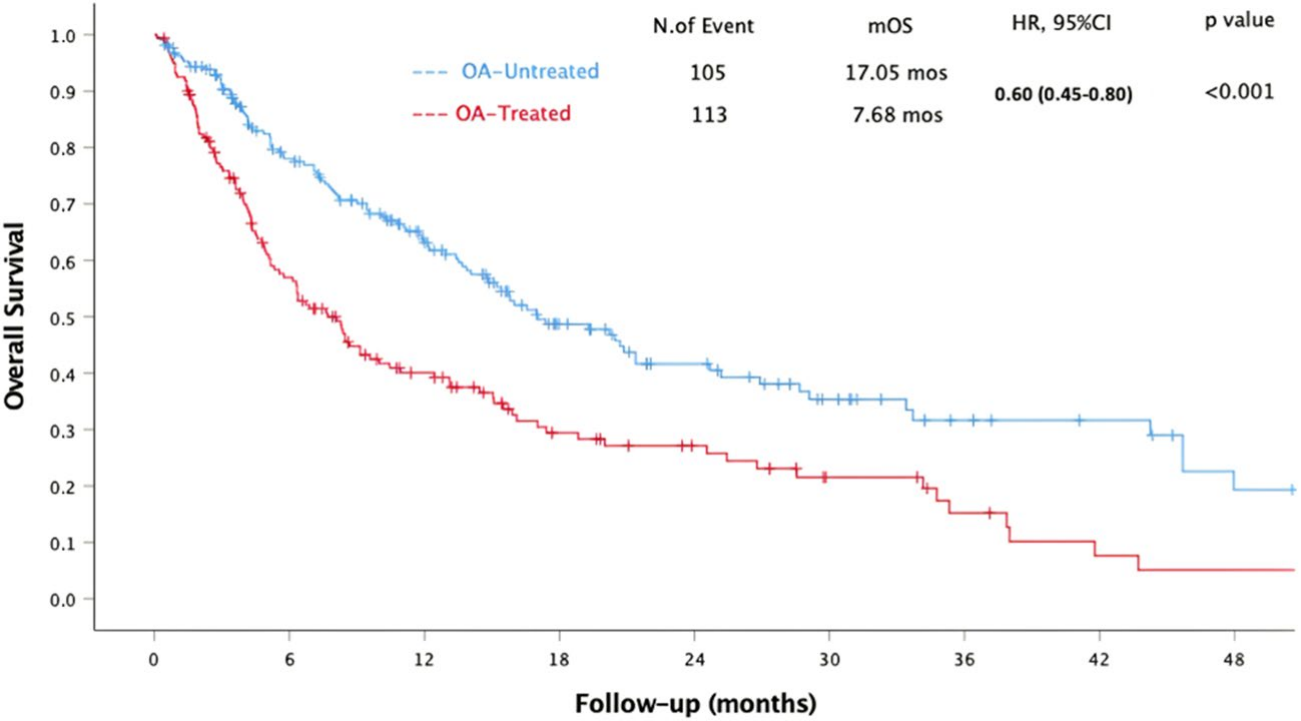
The association between OA treatment and overall survival

The median OS (mOS) was 13.5 months (95% CI 10.81 to 16.19) with 218 deaths. Univariate analyses showed that OA-untreated patients had significantly longer mOS (*p* < 0.001), as did patients with an ECOG PS equal to 0 (*p* = 0.001) and patients with LDH levels within or below the ULN (*p* < 0.001). Although mOS was shorter in patients aged equal to or older than 65 years, the difference was not statistically significant (mOS: 11.07 vs. 15.08, *p* = 0.056). Univariate analysis showed that mOS was significantly longer in RCC (HR 0.47, 95% CI 0.28–0.77, *p* = 0.003), NSCLC (HR 0.51, 95% CI 0.33–0.81, *p* = 0.004), and malignant pleural mesothelioma (MPM) (HR 0.31, 95% CI 0.11–0.90, *p* = 0.031) compared to melanoma. Multivariate analyses confirmed significantly longer mOS in OA-untreated patients (*p* < 0.001), patients with ECOG PS equal to 0 (*p* = 0.016), and patients with LDH levels within or below the ULN (*p* < 0.001). Table 3 summarizes the results of the univariate and multivariate analyses for OS. OA-Untreatment was significantly associated with longer mOS (17.05 vs. 7.68 months, HR 0.60, 95% CI 0.45–0.80, *p* < 0.001), as demonstrated in Fig. 2.

**Table 3:** Univariate and multivariate analyses for overall survival

	Univariate analysis			Multivariate analysis		
	Hazard ratio	95% CI	*p* value	Hazard ratio	95% CI	*p* value
ECOG PS 0 vs. ≥ 1	0.61	0.47–0.81	**0.001**	0.69	0.52–0.93	**0.016**
< vs. ≥ 65 years of age	0.76	0.58–1.00	0.057	0.80	0.58–1.10	0.174
LDH N vs. > ULN	0.52	0.39–0.70	** < 0.001**	0.56	0.42–0.75	** < 0.001**
Primary tumor	0.47	0.28–0.77	**0.020**	0.57	0.33–1.00	0.071
Line of treatment before ICI (3 or more)	1.11	0.80–1.54	0.498			
OA-untreated vs. OA-treated	0.60	0.45–0.80	** < 0.001**	0.58	0.43–0.79	** < 0.001**

*ECOG PS*: Eastern Cooperative Oncology Group performance score, *LDH* lactate dehydrogenase, *ULN* upper limit of the normal range

When evaluated by tumor type, OA treatment was significantly associated with reduced mPFS in patients with melanoma or NSCLC (melanoma: 3.15 vs. 11.07 months, *p* = 0.006; NSCLC: 3.05 vs. 8.14 months, *p* = 0.029), but not in patients with RCC (6.47 vs. 6.24 months, *p* = 0.791). For OS, OA treatment was significantly associated with reduced mOS in patients with melanoma or NSCLC (melanoma: 4.76 vs. 20.2 months, *p* = 0.006; NSCLC: 4.23 vs. 14.85 months, *p* < 0.001), but not in patients with RCC (20 vs. 21.38 months, *p* = 0. 160), head and neck cancer (6.3 months vs. NR, *p* = 0.12), MPM (15.5 vs. 3.8 months, *p* = 0.187), transitional cell carcinoma (6.34 vs. 9.42 months, *p* = 0.417), SCLC (8.08 vs. 7.42, *p* = 0.951), hepatocellular carcinoma (4.3 months vs. NR, *p* = 0.397), and sarcoma (NR vs. 33.4 months, *p* = 0.826).

## Discussion

The use of OAs in cancer patients receiving ICI therapy has become a topic of increasing interest and discussion within the oncology field. This study aims to explore the impact of OAs on survival outcomes in cancer patients undergoing ICI treatment, with a focus on balancing effective pain management and potential immunomodulatory consequences. DDIs have gathered significant attention in the context of identifying patients who benefit from ICI therapy more and who have durable benefits. OAs constitute a significant component of the medications routinely given to cancer patients. Nevertheless, the potential effects of opioids on interactions with ICIs and their role in relieving cancer-related pain are not yet fully comprehended.

Our findings reveal a noteworthy association between OA treatment and significantly reduced PFS and OS in cancer patients receiving ICI therapy. Consistent with previous research hinting at the potential negative influence of opioids, our results highlight that the impact of opioids varies by tumor type, suggesting a need for tumor-specific strategies in clinical practice [6–9]. The relationship between an ECOG PS of 1 or higher and reduced OS, elevated LDH levels above the normal range, and reduced PFS and OS suggests an association between increasing disease burden and OA usage, which may be related to a worse prognosis. Additionally, patients using OA had a higher number of previous treatment regimens, possibly due to underlying factors related to increased disease burden, and this could contribute to decreased survival. This association was particularly noted in malignant melanoma and NSCLC. However, the absence of a similar relationship in RCC and other tumors indicates the potential involvement of different underlying mechanisms. It is also important to consider that the smaller number of patients in other tumor types may impact the interpretation of these results.

Mechanistically, OAs like morphine can impact cancer progression via various pathways. OAs often employed for severe pain management through the activation of the mu (μ) opioid receptors [10] might impact cancer patients’ outcomes during ICI treatment as μ-opioid receptor is also expressed in the cells of the immune system, such as lymphocytes and macrophages [11]. Preclinical investigations have uncovered that opioids such as morphine can trigger tumor proliferation, suppress apoptosis, enhance angiogenesis, and facilitate the invasion of cancer cells through various mechanisms, including EGFR phosphorylation in lung cancer [12], activation of the MAPK/ERK pathway in endothelial cells in breast cancer [13], and induction of urokinase plasminogen activator secretion in colon cancer [5]. Moreover, ICIs’ efficacy depends on anticancer immunity, and opioids may contribute to an immunosuppressive tumor microenvironment. Morphine has been shown to inhibit the transcription of IL-2, a critical cytokine for CD8 + T cell activation, and increase cAMP levels, impairing T-cell receptor signaling and CD8 + T cell function [14]. Additionally, opioids can downregulate major histocompatibility complex class II expression in antigen-presenting cells, inhibiting CD4 + T cell activation and cytokine secretion [15]. Furthermore, opioids can lead to an increase in regulatory Tregs, further hindering the anticancer immune response [16].

Another crucial aspect is the connection between gut dysbiosis and ICI efficacy as imbalances in the gut microbiota can reduce a patient’s response to ICIs [17]. Long-term opioid use, which often results in gastrointestinal side effects, may exacerbate this issue [18]. Opioids can suppress protective mucus and bicarbonate secretion from the intestinal epithelium, weaken myenteric activity, and potentially raise the risk of bacterial translocation [18, 19]. Morphine, in particular, has been found to damage the intestinal epithelial integrity and alter the gut microbiota composition, favoring pathogenic bacteria [18, 20]. These complex interactions underscore the importance of considering the impact of opioids on cancer patients undergoing ICI therapy, urging further research in this field.

Our study, being retrospective in nature, comes with several inherent limitations. It includes a heterogeneous population with variations in primary tumor types, treatment lines, and the types of ICIs administered. Additionally, the OA treatment is more common among patients with high tumor burden and associated symptoms, all of which could potentially act as confounding factors in our retrospective analysis. It was challenging to assess the impact of each specific opioid on survival, as many patients underwent opioid switching during their immunotherapy, often involving different dosages. To address these limitations, future prospective studies should involve larger and more homogeneous patient cohorts while collecting detailed data on opioid dosages, types, and duration of use to validate and expand upon our findings.

Our study provides valuable guidance for oncologists in their clinical practice. It is important to recognize that OAs play an important role in managing cancer-related pain and improving the quality of life for many patients. The decision to prescribe opioids should always be individualized, taking into account the patient’s level of pain, the risk of addiction, and now, the potential impact on cancer outcomes when used with ICIs. To optimize patient outcomes, it is critical to incorporate evidence-based guidelines into clinical practice. Multimodal analgesia, which combines different classes of analgesics and nonpharmacologic interventions, should be prioritized to minimize opioid use and mitigate potential adverse effects. Clinicians should explore alternative pain management strategies, including non-opioid analgesics or interventional procedures, whenever appropriate. Non-opioid analgesics, such as acetaminophen and NSAIDs, should be considered first-line options for mild to moderate pain [21]. For severe pain, adjunctive therapies such as nerve blocks, epidural analgesia, and neuromodulation techniques can provide effective pain relief while reducing the need for opioids [22]. In addition, early integration of palliative care into the treatment continuum has been shown to improve both quality of life and survival outcomes for cancer patients [23]. Palliative care teams can provide comprehensive pain management strategies and psychosocial support to achieve better pain control without relying solely on opioids. Education and training programs for healthcare providers on safe opioid prescribing practices and management of opioid-related side effects are also essential to ensure the safe and effective use of OAs in cancer pain management [24].

In conclusion, the delicate balance between effective pain management and the efficacy of cancer immunotherapy poses a challenging clinical dilemma. Our study provides valuable insights into the potential adverse effects of OAs on survival outcomes in cancer patients undergoing ICI therapy. Clinicians must carefully weigh the benefits of pain relief against the possible negative impact on the immune response and cancer outcomes when considering the use of opioids in this patient population. These findings do not imply the discontinuation of opioid use in cancer patients, as the need for opioids in advanced cancer patients with severe pain is crucial for improving their quality of life. We believe that the primary factor contributing to reduced survival is the high disease burden, but there may also be some additional immunological factors at play, especially considering that we could not observe this relationship in patients with RCC. However, it is essential to emphasize the importance of maintaining this balance. Further research is warranted to better understand the underlying mechanisms and refine clinical guidelines for pain management in cancer patients receiving immunotherapy.

## Conclusion

Our study underscores the importance of careful pain management in cancer immunotherapy. While high disease burden remains the primary factor in reduced survival, emerging evidence highlights immunologic factors and the potential adverse effects of OAs in patients receiving ICIs.

## Data Availability

No datasets were generated or analyzed during the current study.
